# Chicken domestication changes expression of stress-related genes in brain, pituitary and adrenals

**DOI:** 10.1016/j.ynstr.2017.08.002

**Published:** 2017-08-22

**Authors:** Pia Løtvedt, Amir Fallahshahroudi, Lejla Bektic, Jordi Altimiras, Per Jensen

**Affiliations:** AVIAN Behavioural Genomics and Physiology Group, IFM Biology, Linköping University, 58183 Linköping, Sweden

**Keywords:** Animal domestication, Stress response, HPA axis, Glucocorticoid receptor, Gene expression, Chicken

## Abstract

Domesticated species have an attenuated behavioral and physiological stress response compared to their wild counterparts, but the genetic mechanisms underlying this change are not fully understood. We investigated gene expression of a panel of stress response-related genes in five tissues known for their involvement in the stress response: hippocampus, hypothalamus, pituitary, adrenal glands and liver of domesticated White Leghorn chickens and compared it with the wild ancestor of all domesticated breeds, the Red Junglefowl. Gene expression was measured both at baseline and after 45 min of restraint stress. Most of the changes in gene expression related to stress were similar to mammals, with an upregulation of genes such as *FKBP5*, *C-FOS* and *EGR1* in hippocampus and hypothalamus and *StAR*, *MC2R* and *TH* in adrenal glands. We also found a decrease in the expression of *CRHR1* in the pituitary of chickens after stress, which could be involved in negative feedback regulation of the stress response. Furthermore, we observed a downregulation of *EGR1* and C*-FOS* in the pituitary following stress, which could be a potential link between stress and its effects on reproduction and growth in chickens.

We also found changes in the expression of important genes between breeds such as *GR* in the hypothalamus, *POMC* and *PC1* in the pituitary and *CYP11A1* and *HSD3B2* in the adrenal glands. These results suggest that the domesticated White Leghorn may have a higher capacity for negative feedback of the HPA axis, a lower capacity for synthesis of ACTH in the pituitary and a reduced synthesis rate of corticosterone in the adrenal glands compared to Red Junglefowl. All of these findings could explain the attenuated stress response in the domesticated birds.

## 1 Introduction

A number of animal species have adapted to living in the captive environments provided by humans during domestication, allowing the individuals to tolerate proximity to humans, and to live in crowded and confined conditions (Price, 1999). Direct selection by humans, natural selection in the new environment and genetic drift have led to a suite of traits that are commonly associated with domestication, the domesticated phenotype. This includes changes in morphology, physiology and behavior (Price, 1999, Price, 2002) and modifications in the response to stressful stimuli. Stress can be defined as the individual's response to real or perceived threats to homeostasis (McEwen, 2000, McEwen, 2007). The key regulator of the physiological stress response is the hypothalamic-pituitary-adrenal (HPA) axis, with the adrenal glands secreting glucocorticoids into the blood stream.

Glucocorticoids have a wide variety of effects depending on the target tissue, including glycogen breakdown and gluconeogenesis (Exton, 1978, Munk et al., 1984, Coderre et al., 1991, Myers et al., 2014). Their overall function is to shift resource allocation to promote immediate survival, for instance counteracting blood loss and mobilizing energy, while suppressing body functions that are not crucial for immediate survival, such as reproduction, immune system and digestion (Herman et al., 2016). While a stress-induced release of glucocorticoids is beneficial in a short-term challenge, long-term exposure to stress may be harmful to the individual being associated with susceptibility to several diseases, and lowered reproductive ability (De Kloet et al., 2005, Chrousos, 2009). The HPA axis is dependent on a negative feedback system, in which binding of glucocorticoids to glucocorticoid receptors at several levels within the axis can inhibit its activity (De Kloet et al., 2005, Vandenborne et al., 2005, Chrousos, 2009, Keller-Wood, 2011).

The HPA axis has been modified in several different domesticated species in such a way that they have a lower physiological response to acute stress (Weiler et al., 1998, Künzl and Sachser, 1999, Gulevich et al., 2004, Trut et al., 2009, Plyusnina et al., 2011, Soleimani et al., 2011). This modulation may be achieved through modified activity of any of the many proteins involved. A multitude of genes affects this in various tissues, coding for hormones or hormone precursors, receptors, cleaving enzymes, transcription factors and cofactors (Cullinan et al., 1995a, Liu et al., 2013). Any modification in the expression of these genes or the translation of their mRNA to proteins, may lead to changes in the activity of the HPA axis, and highlights possible candidates for explaining domestication effects of the stress response. Upon exposure to an acute stressor, corticotropin-releasing hormone (CRH) and arginine vasopressin (AVP) (in mammals) or arginine vasotocin (in birds) are secreted from the paraventricular nucleus (PVN) of the hypothalamus and transported through the portal vessel to the pituitary (Blas, 2015). Here, CRH stimulates the secretion of adrenocorticotropic hormone (ACTH) into the general circulation. When ACTH reaches the adrenal glands, it initiates the production and release of glucocorticoids, i.e. cortisol, in most mammals and corticosterone in birds and rodents, from the adrenal cortex (Carsia, 2015, Herman et al., 2016).

Additionally, changes in the metabolism of glucocorticoids, mainly taking place in the liver, may also affect the function of the HPA axis (Keller-Wood, 2011). Although there may have been direct selection on stress response and tolerance during domestication, the reduced HPA axis activity observed in domesticated animals may also be partly explained as side effects of selection for other traits due to linkage and pleiotropy (Rauw et al., 1998, Schütz et al., 2004).

The HPA axis in mammals is generally well studied, and much detail is available concerning which genes are involved and how they are controlled (Payne and Hales, 2004, Ellis et al., 2006, Ulrich-Lai and Herman, 2009, Mormede et al., 2011, Herman et al., 2016). However, less is known about the details of the stress response in birds.

Comparing Red Junglefowl (RJF) and domesticated chickens represents an excellent platform for investigating effects of domestication on traits such as the stress response. Domestication of the chicken is thought to have taken place approximately 8000 years ago in South East Asia from a common ancestor, the Red Junglefowl (Tixier-Boichard et al., 2011, Storey et al., 2012, Xiang et al., 2014), still present in its natural habitats in South East Asia.

Investigations of the stress response of these two breeds have previously shown a more fearful behavior, as well as a more pronounced corticosterone increase after acute stress, in RJF (Ericsson et al., 2014, Fallahsharoudi et al., 2015). However, RJF also appear to return to baseline faster than WL, both in terms of behavior and hormonal levels (Ericsson et al., 2014).

Thus, the aim of this study was to assess the effects of domestication on the stress response by monitoring the expression of selected genes involved in the activation and modulation of the HPA axis in five tissues known to be involved in the response in both the ancestral Red Junglefowl and the domesticated White Leghorn breed.

## 2 Methods

### 2.1 Overview of methods

In this project, we used male Red Junglefowl (RJF) and White Leghorns (WL) to investigate the changes in gene expression to an acute stress treatment. At 7 weeks of age, the animals in the stress group were exposed to 45 min of restraint stress. They were then culled, and tissue from hippocampus, hypothalamus, pituitary, adrenals and liver were collected. A group of baseline birds (not exposed to restraint stress) were also culled and sampled for the same tissues. Gene expression analysis with quantitative PCR was then performed on all tissues to compare baseline (unstressed animals) against stress treatment.

Our selection of genes was based both on literature reviews, mainly from rodents, and our previous experiments on chickens. Specifically, in the brain, we focused on genes involved in stimulation and negative feedback of the HPA axis. In the pituitary, we mainly chose genes involved in translating CRH signals to ACTH release. Most of the selected genes in the adrenal glands are involved in steroidogenesis and sympathoadrenal activity, whereas in the liver, we measured genes coding for the corticosterone binding globulin and a gene involved in the metabolism of glucocorticoids.

### 2.2 Ethical statement

All experimental protocols were approved by Linköping Council for Ethical Licensing of Animal Experiments, ethical permit no 50-13. Experiments were conducted in accordance with the approved guidelines.

### 2.3 Animals and housing

We studied one population of domesticated WL and one population of ancestral RJF. The WL population in this experiment (SLU13) was the progeny of an outbred line selected for egg mass and developed for research purposes. The studied RJF population originated from a wild population in Thailand (see (Schütz et al., 2001) for details about the origin of used populations in the experiment). All animals were hatched in our facility and were kept under 12 h light and dark periods with ad libitum access to food and water. The breeds were kept separately in similarly sized (2 m × 2 m) enclosures and similar conditions until they were 6 weeks old when the experiment was conducted. The birds were hatched and kept in groups of around 70 animals, until they were 3 weeks, then they were blood sampled and sex determined. The male birds were then kept in breed-separated pens in groups of around 24 animals.

### 2.4 Sex determination

At the age of three weeks, the chickens were blood sampled from the brachial vein for sex determination. Genomic DNA was extracted from the blood samples according to standard protocols, and sex determination was performed using qPCR based on the method described in Clinton et al. (2001).

### 2.5 Tissue collection

Eight animals from each breed were culled and sampled without going through the stress procedure, whereas eight animals from each breed were culled after 45 min of stress, amounting to a total of 32 birds. Culling was performed by decapitation, and dissection took place immediately after. The top of the skull was opened, and the whole brain was removed. The hippocampal and parahippocampal areas were dissected out from both hemispheres by making a small incision close to the rostral part of the lateral ventricle. Tweezers were then used to carefully pull loose the tissue dorsally and medially to the lateral ventricle, corresponding to the avian hippocampus and parahippocampal area (Puelles, 2007).

Further dissection was performed to dissect a part of the brain enriched in thalamus/hypothalamus from the diencephalon. The pituitary was retrieved by opening the diaphragm sellae and carefully pulling out the tissue from the fossa hypophyseos.

Both of the adrenal glands were dissected out, as well as a part of the liver corresponding to the left lobe adjacent to the colic impression-region. All tissues were immediately frozen in liquid nitrogen and subsequently stored at −80 °C until further processing.

### 2.6 RNA extraction and quantitative PCR

Samples from all 8 baseline individuals from each breed, as well as samples from 8 stress treated individuals from each breed, in total 32 individuals, were used for gene expression measurements. Total RNA was isolated using Ambion TRI Reagent (Life Technologies), according to the supplier's protocol. Depending on the tissue, between 1 and 2 μg total RNA was used for reverse transcription. The reactions were performed with Maxima first strand cDNA synthesis kit for RT-qPCR, with dsDNase (Thermo Fisher Scientific) and a combination of oligo-(dT) 18 primers and random hexamers. We used Primer-BLAST to make primers that were specific to our intended PCR target (Ye et al., 2012). If possible, the primers were designed on exon/exon boundaries to avoid amplification of potential residual genomic DNA. The specificity of each primer was tested by inspecting the melting curve. The PCR was performed in a Light Cycler 480 (Roche Diagnostics, Basel, Switzerland). Each 10 μl reaction contained 1 μl of mixed forward and reverse primers (0.5 μM each), between 40 and 80 picograms cDNA diluted in 4 μl water, and 5 μl SYBR Green I Master (Roche Diagnostics). The following RT-PCR reaction was performed: 5 min 95 °C for activation, followed by 40 cycles (10 s 95 °C, 10 s 60 °C, and 20 s 72 °C). The program was terminated with a melting curve from 72° C to 95° C, and cooling to 40° C. The crossing point values (Cp) were normalized over one or two housekeeping genes (Pfaffl, 2001). Amongst TATA-binding protein, GAPDH, and β2-microglobulin, we chose the ones that showed no difference between breed and treatment and had the least overall variation as housekeeper gene(s) for each tissue were used for normalization.

### 2.7 Statistical analysis

Statistical analyses were performed in R (version 3.1.1), and the plots were made using the ggplot2 R package (Wickham, 2009). To study the effect of breed and stress on normalized gene expression we used linear regression with gene expression as response variable. We first included the potential interaction between treatment and breed (gene expression ∼ breed + treatment + breed × treatment). After correction for multiple testing, no interactions remained significant, and we therefore removed the interaction term from the model. The final model included breed and treatment as fixed predictors (gene expression ∼ breed + treatment). The acquired P-values were adjusted for the total number of tests within each tissue using false discovery rate (Benjamini and Hochberg, 1995).

## 3 Results

Acute stress exposure and breed contributed to the significant effects on expression levels of several genes in each tissue.

**Fig. 1:**
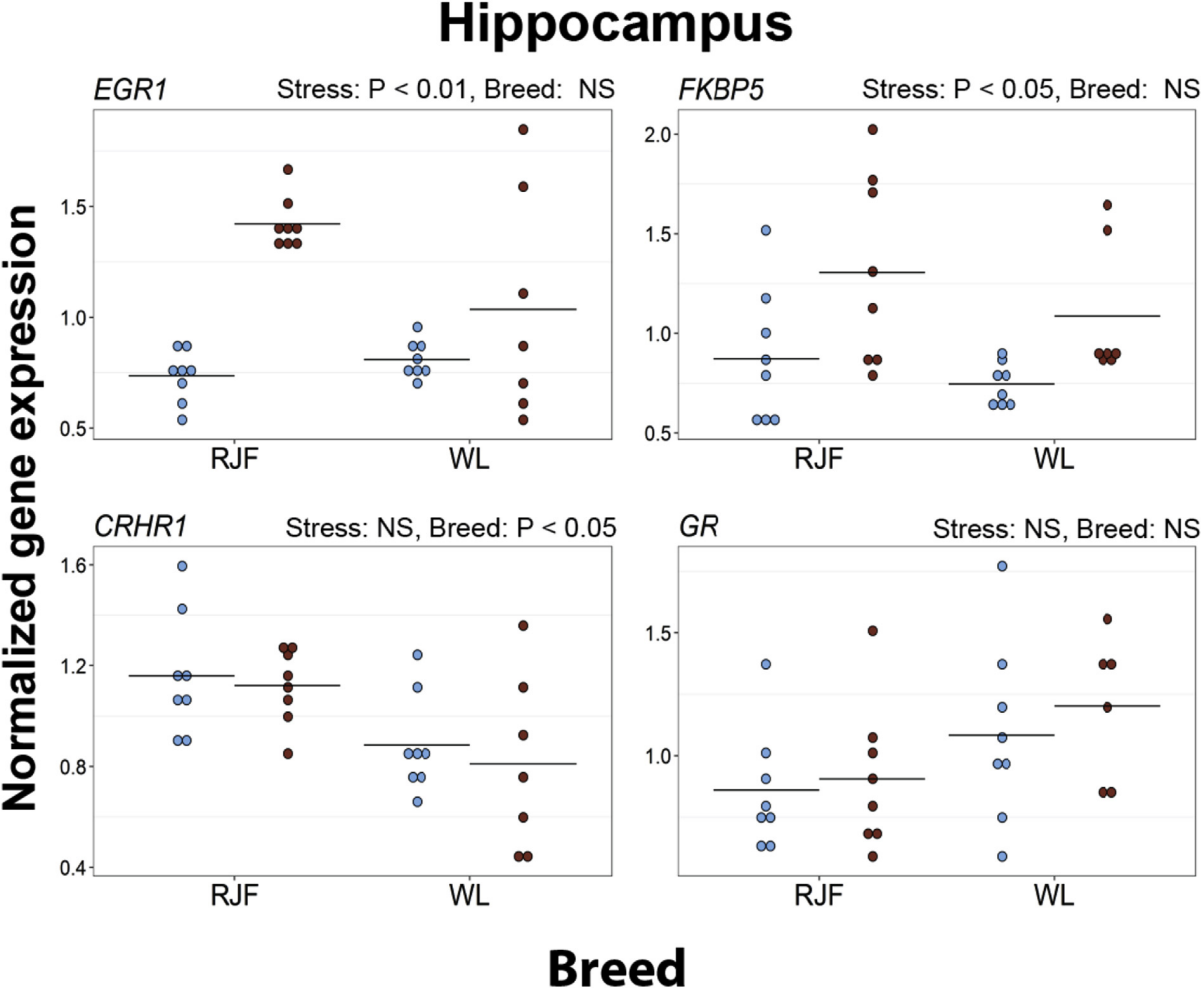
Expression of selected genes in the hippocampal-parahippocampal area of the brain in Red Junglefowl (RJF) and White Leghorns (WL). Mean values are given as horizontal lines, and each individual's value is shown as a marker. Birds sampled at baseline conditions are colored blue (gray on black and white print), and birds sampled after 45 min of restraint stress are colored red (black on black and white print). Genes shown are *early growth response 1* (*EGR1*), *FK506 binding protein 5* (*FKBP5*), *corticosterone-releasing hormone receptor 1* (*CRHR1*) and *glucocorticoid receptor* (*GR*). (For interpretation of the references to colour in this figure legend, the reader is referred to the web version of this article.)

After restraint stress, *EGR1* and *FKBP5* were significantly upregulated in the hippocampus of both breeds. WL had significantly lower expression of *CRHR1* than RJF (Fig. 1 and Table S1).

**Fig. 2:**
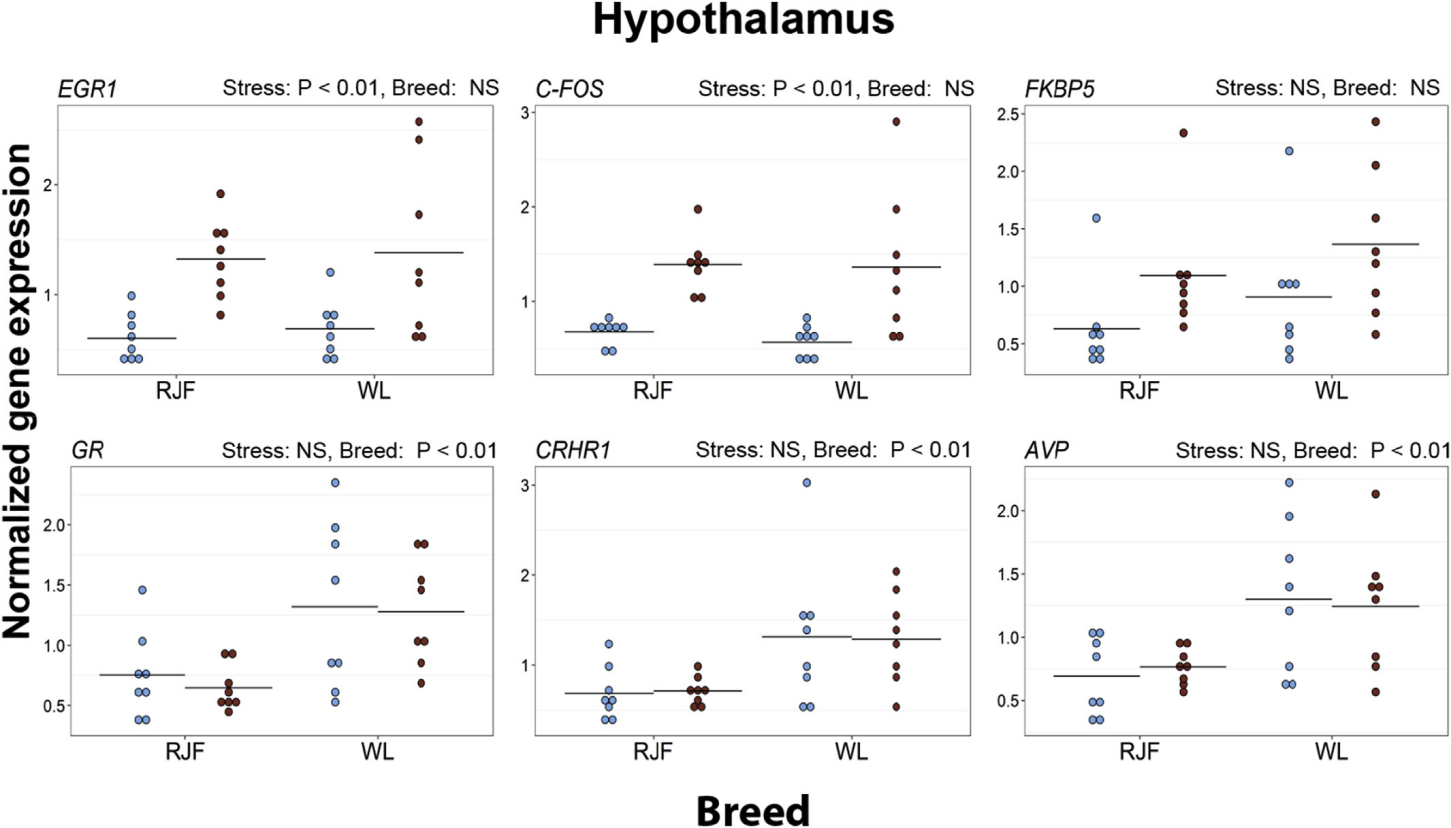
Expression of selected genes in the hypothalamus of Red Junglefowl (RJF) and White Leghorns (WL). Mean values are given as horizontal lines, and each individual's value is shown as a marker. Birds sampled at baseline conditions are colored blue (gray on black and white print), and birds sampled after 45 min of restraint stress are colored red (black on black and white print). Genes shown are *early growth response 1* (*EGR1*), *C-FOS*, *FK506 binding protein 5* (*FKBP5*), *glucocorticoid receptor* (*GR*), *corticosterone-releasing hormone receptor 1* (*CRHR1*) and *arginine vasopressin* (*AVP*). (For interpretation of the references to colour in this figure legend, the reader is referred to the web version of this article.)

In the hypothalamus-enriched tissue, an increase in the expression levels of *EGR1* and *C-FOS* after restraint stress was observed in both breeds. The expression levels of *GR*, *CRHR1* and *AVP* were significantly higher in WL compared with the RJF (Fig. 2 and Table S1).

**Fig. 3:**
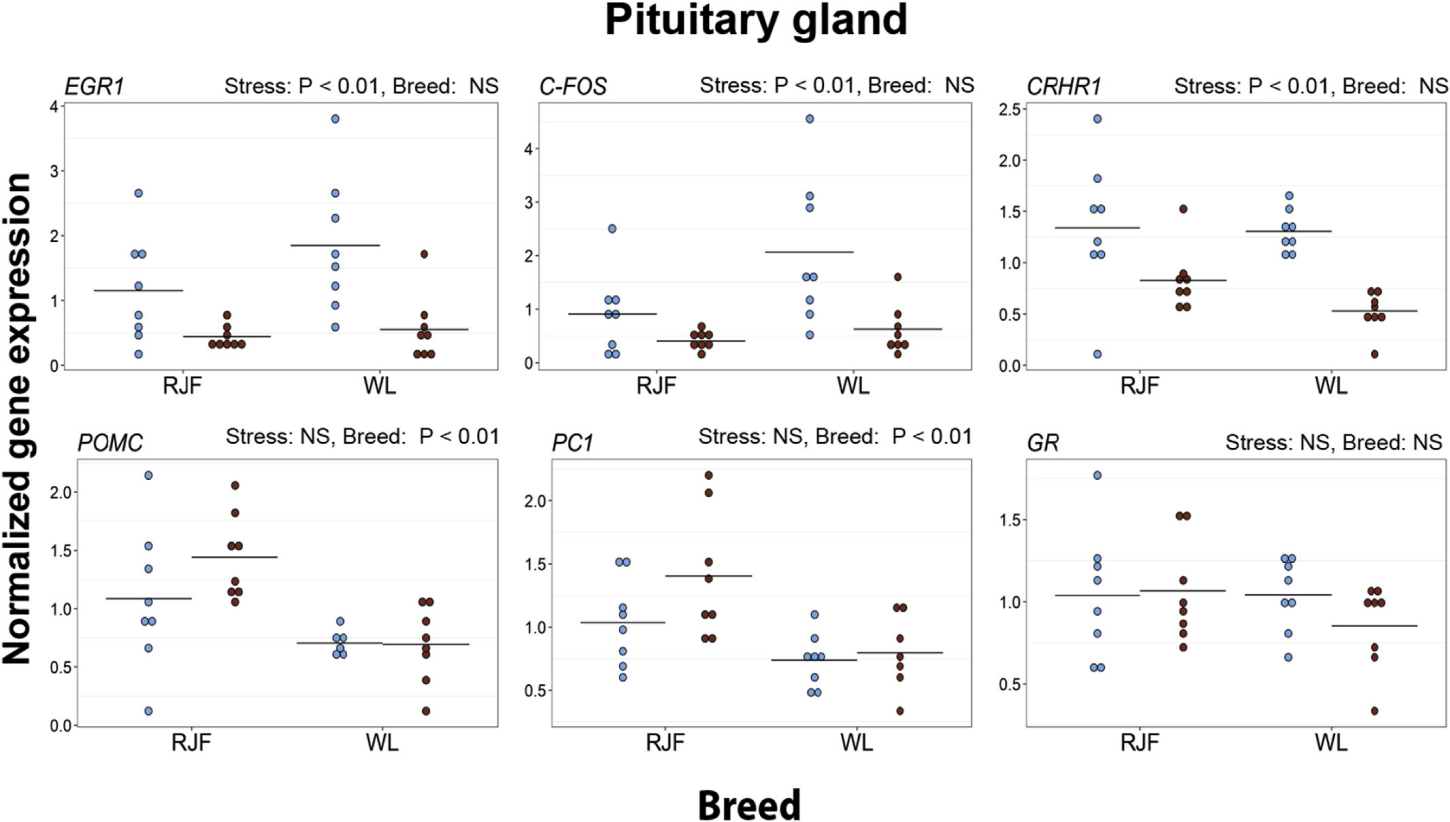
Expression of selected genes in the pituitary in Red Junglefowl (RJF) and White Leghorns (WL). Mean values are given as horizontal lines, and each individual's value is shown as a marker. Birds sampled at baseline conditions are colored blue (gray on black and white print), and birds sampled after 45 min of restraint stress are colored red (black on black and white print). Genes shown are *early growth response 1* (*EGR1*), *C-FOS*, *corticosterone-releasing hormone receptor 1* (*CRHR1*), *proopiomelanocortin* (*POMC*), *proprotein convertase 1* (*PC1*) and *glucocorticoid receptor* (*GR*). (For interpretation of the references to colour in this figure legend, the reader is referred to the web version of this article.)

In the pituitary gland, the expression levels of *EGR1*, *C-FOS*, and *CRHR1* decreased after restraint stress in both breeds. Furthermore, the expression levels of *POMC* and *PC1* were higher in the RJF than in the WL (Fig. 3 and Table S1).

**Fig. 4:**
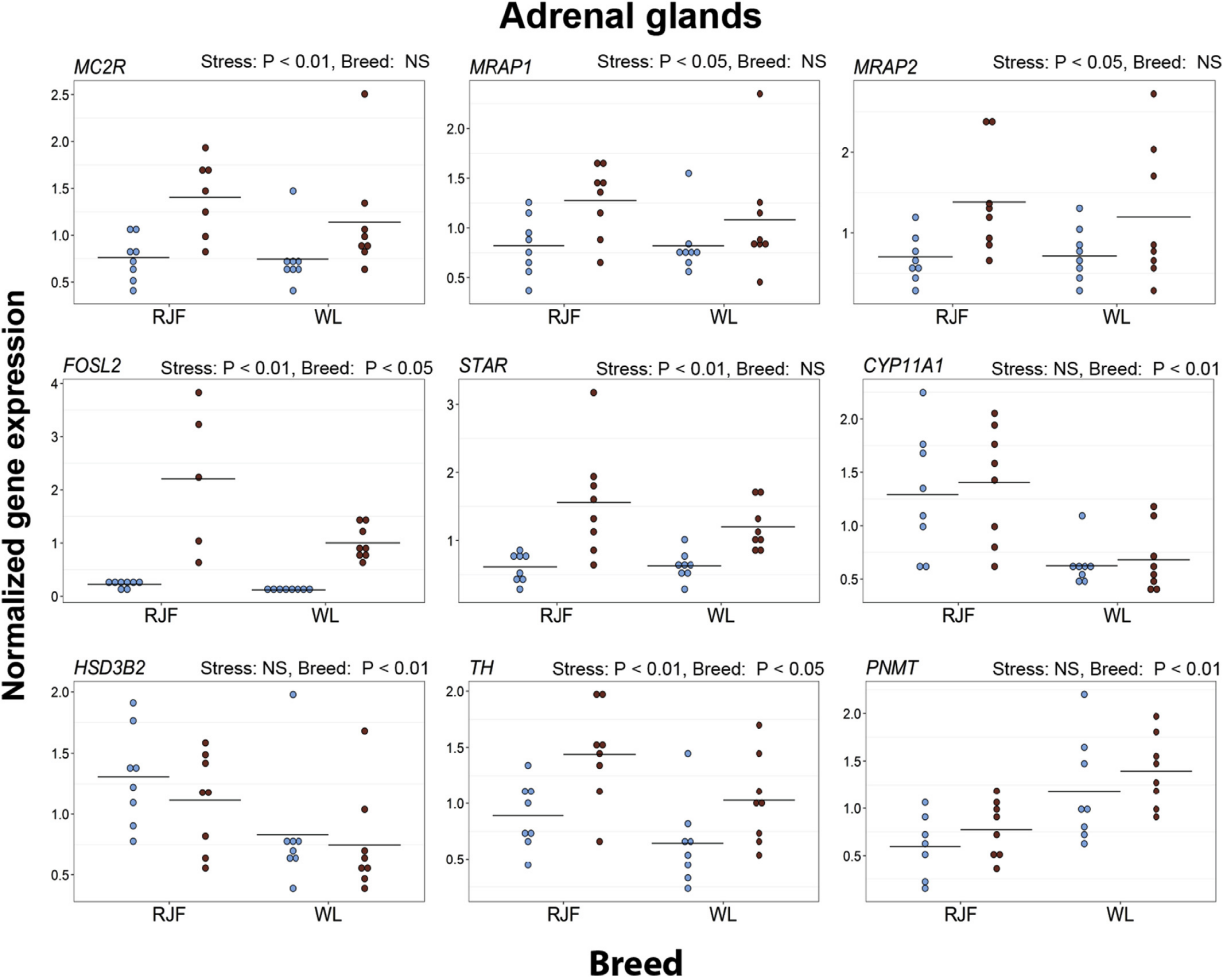
Expression of selected genes in the adrenal glands of Red Junglefowl (RJF) and White Leghorns (WL). Mean values are given as horizontal lines, and each individual's value is shown as a marker. Birds sampled at baseline conditions are colored blue (gray on black and white print), and birds sampled after 45 min of restraint stress are colored red (black on black and white print). Genes shown are melanocortin 2 receptor (*MC2R*), *MC2R accessory protein 2* (*MRAP1*), *MC2R accessory protein 2* (*MRAP2*), *Fos-related antigen 2* (*FOSL2*), *Steroidogenic acute regulatory protein* (*STAR*), *Cytochrome P450 family 11 subfamily A member 1* (*CYP11A1*), *3beta-hydroxysteroid dehydrogenase type II* (*HSD3B2*), *tyrosine hydroxylase* (*TH*) and *phenylethanolamine N-methyltransferase* (*PNMT*). (For interpretation of the references to colour in this figure legend, the reader is referred to the web version of this article.)

In the adrenal glands, the expression levels of *MC2R*, *MC2R* heteronuclear RNA, *MRAP1*, *MRAP2*, *FOSL2, STAR,* and *TH* increased in both breeds after the restraint stress. WL had a higher expression of *CYP11A1* and *PNMT* than RJF, but a lower expression of *HSD3B2*, *FOSL2,* and *TH*. (Fig. 4 and Table S1).

No significant treatment or breed effect was detected for the measured genes in the liver (Table S1).

## 4 Discussion

Our data are the first to throw light on the detailed genetic control of the stress reaction in an avian species, and to investigate the mechanisms of the domestication induced modified HPA response in chickens. We report changes in the expression of numerous stress-related genes in chickens upon an acute stress exposure and we show that some of these changes differ between the ancestral RJF and the domesticated WL. This pattern was observed in different tissues, which highlights multiple regulation in the HPA-axis and suggests that domestication has changed the stress response of chickens by a complex modification of gene expression. In particular, we found that the combination of a higher expression of *GR* in hypothalamus following stress in domesticated WL, a lowered *POMC* gene expression in pituitary and a decreased expression of steroidogenic genes in the adrenal glands could explain the hampered HPA axis reactivity in WL compared to ancestral RJF.

### 4.1 Stress effects on gene expression

In all investigated tissues except from the liver, we found differences in gene expression between birds sampled at baseline and those sampled after 45 min of stress.

In the hippocampus, expression levels of *EGR1* and *FKBP5* increased after the stress treatment. FKBP5 is involved in an ultra-short negative feedback loop for GR sensitivity. FKBP5 expression is induced by corticosterone binding to GR, and in turn, higher levels of FKBP5 cause a decrease in GR sensitivity to corticosterone (Binder, 2009). In rats, it has been shown that exposure to a variety of stressors can lead to upregulation of *FKBP5* in the hypothalamus and to a lesser extent in the hippocampus (Scharf et al., 2011). However, in our study, the effect of stress on mRNA levels of *FKBP5* was not significant in the hypothalamus after correction for multiple testing (Table S1).

The *EGR1* gene encodes an immediate-early gene, which is upregulated in many tissues after neuronal input (Cullinan et al., 1995a) including the avian hippocampus (Grella et al., 2016). Both *EGR1*, and another immediate-early gene, *C-FOS*, were upregulated after stress treatment in the hypothalamus. An upregulation of these genes in response to stress has been documented in several brain areas (Cullinan et al., 1995a), and we can therefore conclude, together with our hormonal data, that our stress treatment had a marked effect on the chickens.

Contrary to our expectations, we did not detect higher expression of *POMC*, *CRH*, and *AVP* after stress in the hypothalamus, despite previous reports of these genes being upregulated after stress in rats and mice (Lightman and Young, 1988, Givalois et al., 2000, Lee et al., 2005). However, in birds the timeline of gene expression alterations and protein level changes after stress exposure is not well known. While the stressor we used may be considered quite severe, it is still possible that the rapid feedback control of the HPA axis has caused expression levels to return to baseline again at the sampling point. More investigations about the time course of gene expression after immediate stress is needed before any final conclusion can be made.

In the pituitary gland, expression levels of *CRHR1* decreased in both breeds after 45 min of restraint stress. CRHR1, a receptor for CRH, is an important link between CRH release from the hypothalamus and the secretion of ACTH from the pituitary (Scanes, 2015). Downregulation of *CRHR1* in the pituitary may decrease the release of ACTH and hence, may have an important function on regulation of the HPA axis in chickens. This downregulation of *CRHR1* has also been reported in rodents (Vandenborne et al., 2005), and it is mainly mediated by the action of a microRNA (Nemoto et al., 2013).

We found a decrease in the expression of *EGR1* and *C-FOS* after stress in the pituitary. This is the first report investigating these genes in the chicken pituitary after stress, and more investigations are needed to understand the exact role that this downregulation has. However, *EGR1* in the pituitary is involved in the production and the release of luteinizing hormone (LH) in mammals through stimulating the production of LH subunit β, and a lack of *EGR1* expression causes a reduction in LH levels (Wolfe and Call, 1999; Tremblay and Drouin, 1999. The expression of *EGR1* in the pituitary is regulated by hypothalamic gonadotropin-releasing hormone (GnRH) (Ferris and Shupnik, 2006, Burger et al., 2009), and GnRH production is in turn inhibited by CRH (Gambacciani et al., 1986, Rivier and Rivest, 1991, Chrousos et al., 1998). We have previously shown that plasma levels of several sex steroids decreased already one hour after restraint stress (Ericsson et al., 2014), and this change in *EGR1* could be a mechanism linking the stress exposure to this decrease.

To our knowledge, a decreased expression of *C-FOS* in the pituitary after stress has not been reported in any species before, although an increase of *C-FOS* expression in the pituitary of male rats after 20 min of novelty stress has been found (Handa et al., 1993). The expression of *C-FOS* may be stimulated by growth hormone-releasing hormone (Billestrup et al., 1987), and in turn, growth hormone release is inhibited during stress (Tsigos and Chrousos, 2002). C-FOS could thus potentially be involved in the stress-related decrease of growth hormone, although more work is needed to clarify its role in the pituitary.

The adrenal glands produce the majority of circulating adrenaline and glucocorticoids (Carsia, 2015). We measured expression of the genes coding for proteins mediating different steps in the production of both glucocorticosteroids and catecholamines. The expression levels of *MC2R*, *MC2R* heteronuclear RNA, *MRAP*, *MRAP2, STAR*, *FOS-L2* and *TH* were higher in the samples collected after 45 min of restraint stress in both breeds. Similar patterns of gene expression have been reported in the adrenal glands of mammals after stress (McMahon et al., 1992, Cullinan et al., 1995b, Sabban and Kvetňanský, 2001, Stocco, 2001, Mormede et al., 2011). Overall, this indicates that specific functioning of the steroidogenesis is rather conserved between mammals and avian species.

In the liver, we saw no changes in gene expression of the investigated genes: serpin family A member 10 (*SERPINA10*), serpin family A member 4 (*SERPINA4*) and hydroxysteroid 11-beta dehydrogenase 2 (*HSD11B2*) after stress. *SERPINA10* was recently identified as a candidate gene for domestication effects on the function of the HPA axis in chickens (Fallahsharoudi et al., 2016), the gene currently annotated as *SERPINA4* has been identified as the gene encoding corticosterone-binding globulin (CBG) (Vashchenko et al., 2016), while the enzyme HSD11B2 converts corticosterone to inactive cortisone (Carsia, 2015). The lack of any effect of stress treatment on these genes suggests that neither the removal of glucocorticosteroids from the blood, nor the amount of CBG present in the blood are affected by acute stress.

### 4.2 Domestication effects on gene expression

An important goal of this study was to investigate gene expression differences between domestic and ancestral chickens in relation to the stress response. Several interesting differences in expression levels were found in all investigated tissues but the liver.

In the hippocampus, the expression of *CRHR1* was higher in RJF than in WL. CRH, apart from being an important hormone in the HPA axis, is also a neurotransmitter released in many areas of the brain, with a variety of different functions (Heinrichs and Koob, 2004). In the hippocampus, the CRH system is involved in the storage of fear memories (Chen et al., 2012) and increased *CRHR1* levels in RJF in this part of the brain may indicate differences in fear memory handling in the two breeds. Studies in mice have also shown that forebrain deletion of CRHR1 causes some resistance to adverse effects from chronic stress (Heinrichs and Koob, 2004, Wang et al., 2011a, Wang et al., 2011b).

Expression levels of *GR*, *CRHR1*, and *AVP* were all higher in the hypothalamus-enriched region of WL than RJF. The hypothalamus is an important brain area for negative feedback of the HPA axis (De Kloet et al., 2005, Keller-Wood, 2011). Modulation of the negative feedback mechanism may take place through for example changes in the numbers of receptors available for binding to glucocorticoids, and such changes to the feedback system may have large effects on the timing of glucocorticoid release as well as the magnitude of the peak levels. Higher levels of GR in the hypothalamus may thus underlie the cascade of observed differences in the transcription of downstream genes and ultimately explain the hampered HPA axis reactivity in the domesticated chicken. It may be worth noting that the expression levels of *GR* in hippocampus followed the same pattern as in the hypothalamus, but the difference between the breeds is smaller in hippocampus and does not remain statistically significant after correction for multiple testing. The levels of pituitary *POMC* and *PC1* were higher in the RJF than the domesticated WL. POMC is the precursor of ACTH, while PC1 is involved in the cleavage of POMC into ACTH (Karpac et al., 2007, Keller-Wood, 2011). The lower expression levels of POMC and PC1 in the pituitary of WL, indicating an attenuated production of ACTH, may be linked to the lowered HPA axis reactivity generally found in the domesticated chicken (Soleimani et al., 2011, Ericsson et al., 2014, Fallahsharoudi et al., 2015).

In the adrenal glands, CYP11A1 and HSD3B2 are the key enzymes that convert cholesterol into pregnenolone and ultimately into glucocorticosteroids (McMahon et al., 1992, Lin et al., 1995, Sewer and Waterman, 2003, Xing et al., 2010). The expression levels of both *CYP11A1* and *HSD3B2* were higher in the RJF compared with the domesticated WL. The differences in the levels of these steroidogenic genes in the adrenal gland may result in a lower production of corticosterone in the domesticated chicken.

The lack of any breed differences in expression of *MC2R* and the genes encoding its accessory proteins *MRAP* and *MRAP2* suggests that the sensitivity of the adrenal glands to ACTH has not been modified by domestication.

The lack of any differences between the breeds in the genes investigated in the liver suggests that the two breeds have both a similar removal rate of corticosterone and similar carrying capacities of corticosterone in the blood by CBG.

Taken together, the expression differences we find between these two breeds at various levels of the HPA axis are in line with previous observations that domesticated animals, including WL, have an attenuated stress response compared to their wild counterparts. Particularly interesting are the findings of higher *POMC* and *PC1* expression in the pituitary and higher *CYP11A1* and *HSD3B2* in the adrenals of RJF, which suggest a higher production of ACTH and corticosterone respectively. The lack of any interaction between breed and treatment on gene expression suggests that modification of the HPA axis throughout domestication has only affected overall expression levels of the measured genes.

While we have found numerous gene expression differences between the two breeds in the HPA axis, we do not yet know if these are all caused primarily through changes in one or a few genes, followed by a cascade of downstream pathway genes. One potential cascade initiator could be the observed higher expression levels of the GR in hippocampus and hypothalamus of WL. This may have orchestrated the changes in expression levels of *POMC* in the pituitary and ultimately downregulated expression of *HSD3B2* and *CYP11A1* in the adrenal glands (Sewer and Waterman, 2003, Liu et al., 2008). Another possibility is that independent regulatory elements are responsible for gene expression in each tissue. Investigating the ontogeny of observed differences in gene expression in the different tissues might provide further insights. Another interesting next step would be to perform a larger analysis on some of these tissues to gain more information about less well known genes that could influence the stress response. In particular, a study involving RNA sequencing or microarray investigations on the gene expression pattern of the pituitary gland could provide extensive novel information.

Domesticated animals are exposed to a range of acute stressors such as handling as well as chronic stressors like living in a crowded environment and the presence of predators' odor. The focus of our study was only the acute stress response and its modification during chicken domestication. However, the negative effects of chronic stress on welfare of farm animals are well documented (Rauw et al., 1998) and hence, a different study design may address the potential role of animal domestication on stress resilience in chicken.

## 5 Conclusion

In conclusion, our data suggest that the overall pattern of gene expression after acute stress in chickens is similar to that of mammals. Furthermore, domestication of chickens has affected the expression of a wide array of genes of importance on several levels in regulation of the HPA-axis. Higher expression levels of *GR* in hypothalamus combined with lowered *POMC* gene expression in pituitary and lowered expression of steroidogenic genes in the adrenal glands suggest that the domesticated White Leghorn may have a higher capacity for negative feedback of the HPA axis, a lower production capacity for ACTH in the pituitary and a reduced synthesis rate of corticosterone in the adrenal glands compared to Red Junglefowl. All of these findings could explain the attenuated acute stress response in the domesticated birds. The study show how domestication has altered the stress response to make chickens more resilient to acute stress, and that this may be true for many domestic animals. Our results also give important insights into the very limited knowledge on the physiological stress response in avian species, and the findings can have importance for both science and the poultry industry.
