# Supplementary material for: Chicken domestication changes expression of stress-related genes in brain, pituitary and adrenals
Source: Neurobiol Stress. 2017 Aug 22;7:113–21. doi: 10.1016/j.ynstr.2017.08.002 (PMC5577413; doi:10.1016/j.ynstr.2017.08.002)
Supplement: Supplementary Table 1 [file mmc1.pdf]

Supplementary Table 1

Tissue	Gene	Breed			Treatment		
		t	P	adj. P	t	P	adj. P
Hippocampus	*EGR1*				4,61	0,00	0,00
	GR	2,33	0,03	0,138			
	AVP						
	AVPR1A						
	FKBP5				3,20	0,00	0,02
	MR						
	BDNF						
	CRHR2						
	CRH						
	CRHR1	-3,44	0,00	0,018			
Hypothalamus	*POMC hnRNA*						
	AVP hnRNA						
	GR	3,75	0,00	0,007			
	AVP	3,65	0,00	0,007			
	FKBP5				2,45	0,02	0,10
	AVPR1A						
	POMC						
	CRH						
	CRHR2						
	CRHR1	3,43	0,00	0,009			
	C-FOS				5,10	0,00	0,00
	EGR1				4,43	0,00	0,00
	TH						
	BDNF						
Pituitary	*AVPR1B*				2,06	0,05	0,12
	POMC hnRNA						
	GR						
	EGR1				-3,95	0,00	0,00
	C-FOS	2,32	0,03	0,081	-3,27	0,00	0,01
	AVPR1A						
	POMC	-3,85	0,00	0,007			
	CRHR2	2,25	0,03	0,081			
	PC1	-3,47	0,00	0,008			
	CRHR1				-4,62	0,00	0,00
Adrenal gland	*MRAP2*				2,85	0,01	0,02
	MRAP1				2,57	0,02	0,03
	MC2R				3,49	0,00	0,01
	STAR hnRNA				2,16	0,04	0,06
	HSD3B2	-2,86	0,01	0,034			
	FOSL2	-2,48	0,02	0,052	5,78	0,00	0,00
	CYP11A1	-4,61	0,00	0,001			
	MC2R hnRNA				2,32	0,03	0,05
	DBH						
	STAR				4,67	0,00	0,00
	PNMT	4,26	0,00	0,001			
	TH	-2,47	0,02	0,052	3,49	0,00	0,01
	CYP11A hnRNA	-2,27	0,03	0,067			

Results from linear regression with gene expression as response variable and breed and treatment as fixed predictors (gene expression ~ breed + treatment). The acquired P-values were adjusted for the total number of tests within each tissue using false discovery rate (FDR).
